# Unusual Presentation of Tuberculous Thyroid Abscess in a Background of Hashimoto's Thyroiditis in a Chronic Hepatitis B Carrier

**DOI:** 10.1155/2016/5295236

**Published:** 2016-12-22

**Authors:** Sakthivel Chinnakkulam Kandhasamy, Sunil Kumar, Anubhav Sangwan, Neelam Sahani, Gopalakrishnan Gunasekaran, Sunil Kumar Meena, Swapnil Singh Kushwaha

**Affiliations:** ^1^Department of General Surgery, Vardhman Mahavir Medical College and Safdarjung Hospital, New Delhi, India; ^2^Department of Pathology, Vardhman Mahavir Medical College and Safdarjung Hospital, New Delhi, India

## Abstract

Tuberculosis of thyroid gland is a very rare disease. It has variable presentations and may be sometimes associated with autoimmune thyroiditis. We report a case of 45-year-old male, with left sided painless neck swelling, with a purulent discharging sinus over it associated with night sweats and loss of appetite. Thyroid imaging disclosed heterogeneous enhancement of left lobe of thyroid gland with internal vascularity and coarse calcifications. Core needle biopsy revealed caseous necrosis and AFB positivity. Patient had thyroid peroxidase antibody and thyroglobulin antibody positivity and the rest of thyroid function tests were normal. Patient had positive Mantoux test, hepatitis B surface Ag, and low viral DNA. The patient was diagnosed as being a case of tuberculous abscess of thyroid gland and was put on antitubercular therapy for 2 months. Patient subsequently underwent left hemithyroidectomy when there was no response. Histopathological examination revealed tuberculosis of thyroid gland. A final diagnosis of tuberculous abscess of thyroid gland in a background of Hashimoto's thyroiditis in a chronic HBV carrier was made. Therefore, although rare tuberculosis of thyroid should be kept in mind as a differential diagnosis of thyroid swelling.

## 1. Introduction 

Tuberculosis of thyroid gland is extremely uncommon even in countries where the prevalence of tuberculosis is high. The exact number of cases reported is difficult to estimate; barely 200 cases can be found in literature [1]. The mycobacterium tuberculosis reaches the gland through blood and lymphatics or direct extension from cervical lymph nodes. It has variable presentation, but most cases manifest as a nodule or caseous abscess. The diagnosis can be made from FNAC (fine needle aspiration cytology) and culture of the aspirated material or sometimes by histopathological examination of resected gland.

Hashimoto's thyroiditis also known as chronic autoimmune lymphocytic thyroiditis is the most common autoimmune thyroid disease. It is multifaceted disease exhibiting various clinical presentations. It presents with painless enlargement of the thyroid gland and is associated with detection of thyroid autoantibodies [2]. Literature shows that the disease is 15 times more common in women with the peak incidence varying between the ages of 30 and 50 [2, 3]. Environmental triggers, drugs, chemicals, and infection contribute to autoimmune thyroid disease. Thyroid involvement may be regarded as the most frequent alteration in HCV (hepatitis C virus) positive patients and is more frequent than in HBV (hepatitis B virus) positive patients. Thyroid autoimmunity in viral infection may be due to a cytokine-induced disease in susceptible patients [4].

We here present a case of 45-year-old male with tuberculous thyroid abscess in a background of Hashimoto's thyroiditis in a chronic hepatitis B carrier.

## 2. Case Presentation

**Figure 1:**
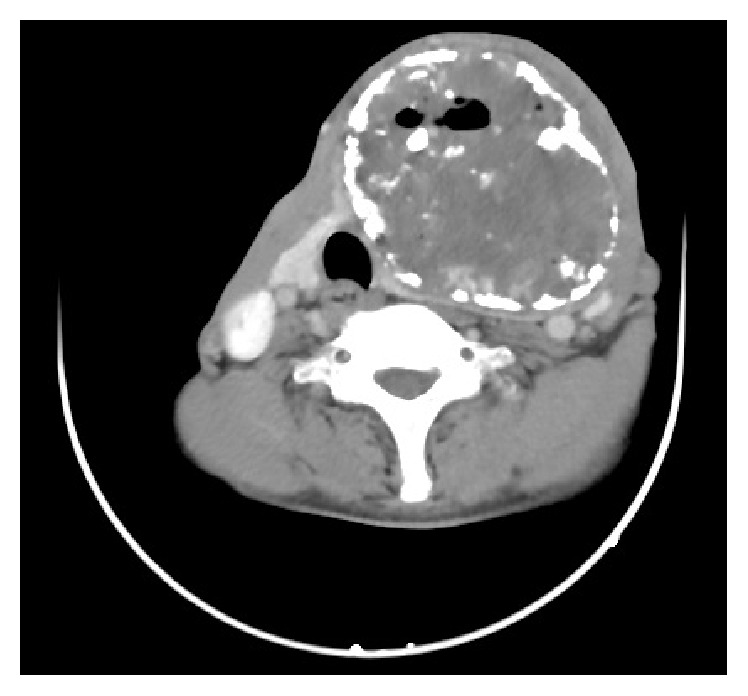
Computed tomography revealing enlarged left thyroid lobe with heterogeneous enhancement and calcifications. There was a hypodense component with focal air loculi within the lesion.

**Figure 2:**
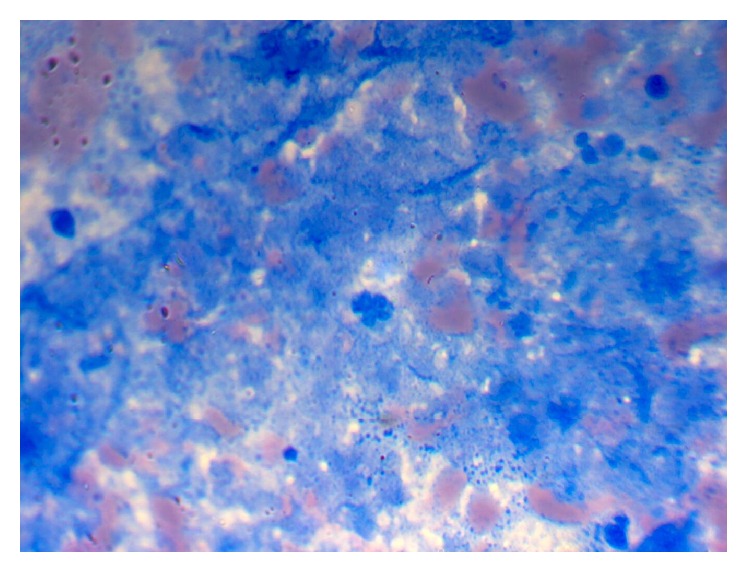
Core needle biopsy showing caseous necrosis and stain for AFB positive.

A 45-years-old male, with no significant past medical history, presented to the outpatient department with 15-year history of painless neck swelling in the left side and 5-month history of purulent discharging sinus over it. The swelling was slowly progressive in nature and he had mild discomfort while swallowing. There was no complaint of dyspnea or hoarseness of voice. There was no specific history suggestive of hypo- or hyperthyroidism. Though the patient denied any history of fever, he complained of night sweats and loss of appetite. Patient did not have any past history or family history of tuberculosis. Physical examination revealed enlarged left lobe of thyroid (10 × 8 cm) with a single purulent discharging sinus over it with undermined edges. There were no palpable cervical lymph nodes. Pulmonary system examination was normal. Thyroid hormone assay revealed thyroid stimulating hormone (TSH) 2.99 *μ*IU/mL, thyroxine (T4) 9.08 *μ*g/dL, triiodothyronine (T3) 138 ng/dL, thyroglobulin (Tg) 42 ng/mL, thyroglobulin antibody (TgAb) 150 IU/mL, and thyroid peroxidase antibody (TPOAb) 232 IU/mL. Hematological examination revealed hemoglobin 12 g%, hematocrit 42%, ESR 75 mm/first hour, platelets 196000/mm^3^, and WBC 9300/mm^3^, with normal differential count. Chest X-ray was normal but Mantoux test was positive (20 × 18 mm). Viral markers test for HIV and anti-HCV was negative. But patient was positive for HbsAg with HbeAg negative and hepatitis B DNA titre of 799 IU/mL. Thyroid ultrasonography revealed enlarged left lobe of thyroid with heterogeneous contents with calcifications. Contrast enhanced computed tomography was performed which showed heterogeneous enhancement of the left lobe of thyroid gland (79 × 95 × 88 mm) with internal vascularity and coarse calcifications. There was a hypodense component with focal air loculi within the lesion (Figure 1). FNAC showed focal necrosis against hemorrhagic background without demonstrable acid fast bacillus (AFB). Culture of the aspirated fluid for mycobacterium tuberculosis came out negative. Core needle biopsy was performed which showed severe chronic inflammation, caseous necrosis, and stain for AFB positive (Figure 2). The patient was put on WHO category 1 antitubercular regime, that is, combination of rifampicin 450 mg, isoniazid 300 mg, ethambutol 1200 mg, and pyrazinamide 1500 mg thrice a week for 2 months. After 2 months, patient had developed progression of swelling with pressure symptoms for which he underwent left hemithyroidectomy.

**Figure 3:**
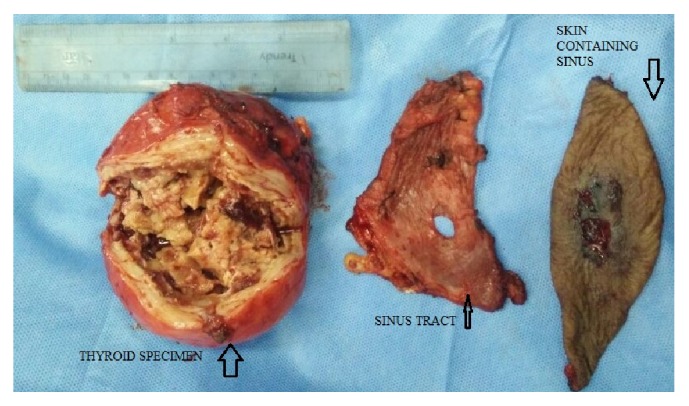
Cut section of thyroid showing greyish white color with extensive areas of autolysis and a cavity identified in the center of the lesion.

**Figure 4:**
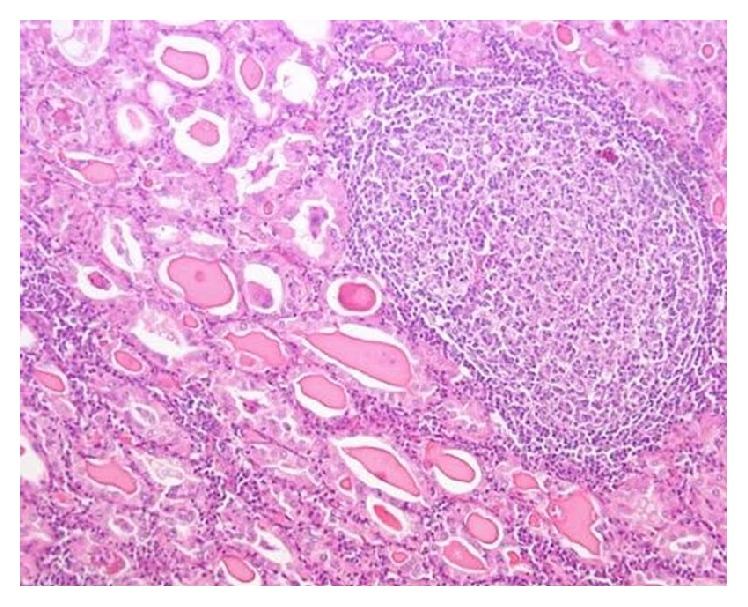
Thyroid histopathology showing destruction of thyroid follicles. Numerous lymphoid follicles were seen with lymphocytes infiltrating into the surrounding fibrocollagenous tissue.

**Figure 5:**
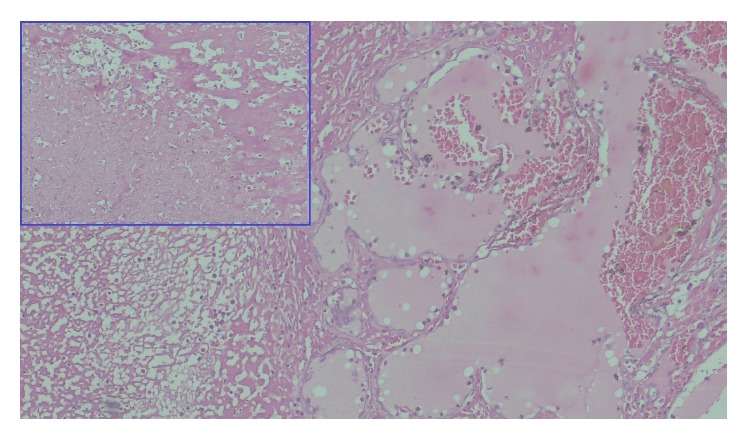
Tissue section showing areas of necrosis and adjacent thyroid follicles suggestive of tuberculosis of thyroid gland. Boxed area showing extensive areas of necrosis.

Grossly, the left thyroid lobe was enlarged measuring 8 × 9 × 8 cm. The cut section was greyish white with extensive areas of autolysis and a cavity identified in the center of the lesion (Figure 3). On histopathological examination, there was destruction of thyroid follicles and extensive fibrosis at the periphery. Numerous lymphoid follicles were seen with lymphocytes infiltrating into the surrounding fibrocollagenous tissue. A 1 cm sinus tract was identified which was lined by dense inflammatory granulation tissue and fibrosis, features suggestive of Hashimoto's thyroiditis (Figure 4) and areas of extensive necrosis adjacent to thyroid follicles suggestive of tuberculosis of thyroid (Figure 5). Final diagnosis of tuberculous thyroid abscess in a background of Hashimoto's thyroiditis in a chronic hepatitis B carrier was made. Postoperatively patient was continued on antituberculous regimen for 9 months.

## 3. Discussion

Thyroid tuberculosis is an extremely rare, whether primary or secondary. Certain tissue in our body is resistant to tuberculosis including thyroid, cardiac muscle, skeletal muscle, and pancreas [5]. Thyroid gland is known to resist the infection but exact reason remains unclear. It is assumed that various reasons included the presence of bactericidal action of colloid material, thyroid capsule, antitubercular action of thyroid hormones, increased blood flow, and increased iodine which cause destruction of tubercle bacilli [6].

The incidence of thyroid tuberculosis is 0.1% to 0.6% in resected specimens [7]. Clinical presentation is variable and symptoms are often nonspecific. The patient may present with goitre and associated pressure symptoms like dyspnea, dysphagia, and hoarseness of voice. There may be a painless discharging sinus. Diagnosis can be made from FNAC with staining for AFB or culture of the aspirated material. The characteristic histological features include caseous necrosis, necrotizing epithelioid granuloma, and Langhans type giant cells. Demonstration of acid fast bacilli by Ziehl-Neelsen staining confirms the diagnosis but it is often negative in tissue section [8]. Thyroid tuberculosis must be differentiated from other granulomatous disorders. Sometimes patient may present with subacute granulomatous (De Quervain's) thyroiditis or chronic thyroiditis [9]. Our patient 45-year-old male who was a chronic hepatitis B carrier presented with tuberculous thyroid abscess in a background of Hashimoto's thyroiditis.

Previously, treatment of thyroid tuberculosis consisted of antituberculous drug and surgical removal of the gland [10]. Lately it has been found that antituberculous drug alone is sufficient to control the disease [11]. If the patient presents with large abscess, surgical drainage or resection is often necessary [12].

Hashimoto's thyroiditis is one of the most common thyroid disorders. The disease has been called Hashimoto's thyroiditis, chronic thyroiditis, lymphadenoid goitre, and recently autoimmune thyroiditis. In this disease there will be autoimmune destruction of thyroid glands associated with chronic lymphocyte infiltration, eosinophilic change, fibrosis, and atrophy of the thyroid gland. Patient may present with painless diffuse enlargement of thyroid gland. At times, there will be no goitre at presentation. It may be associated with hypothyroidism [2, 3].

In autoimmune thyroiditis, there is presence of thyroid autoantibodies in patients' sera against two major thyroid antigens (thyroid peroxidase and thyroglobulin). Thyroid peroxidase (TPO) is essential for various steps in thyroid hormone synthesis, iodine oxidation, iodination of tyrosine residues, and coupling of iodotyrosine. TPO is located at the apical membrane of the thyroid follicles. Thyroglobulin (Tg) is located within the follicles and helps in storage of thyroid hormones [13]. Antibodies against TPO (TPOAb) and Tg (TgAb) are able to cause destruction of thyroid cells due to antibody dependent cell cytotoxicity. These antibodies are useful in the diagnosis of thyroid autoimmunity. In Hashimoto's thyroiditis, TPOAbs are present in nearly all (>90%) patients, while TgAbs can be found in 80% [14, 15].

Viral infections play a role in the development of autoimmunity possibly by the molecular mimicry between viral and self-antigens. Viral infection can cause the release of proinflammatory mediators which may lead to activation of autoreactive T-cells [13]. Autoimmune phenomenon is more common with hepatitis C virus (HCV) than hepatitis B virus (HBV). Therapy with interferon has important effects on expression of major histocompatibility antigens [16] and the regulation of cytokine production [17]. Autoimmunity against the thyroid gland in patients with hepatitis B infection is not well understood. Positive levels of TPOAb and TgAb were found in 20% and 11% of patients with HCV compared with 5% and 3% of patients with HBV, respectively [4]. Variable geographic distribution has also shown that genetic or environmental influences could be implicated [18]. On the whole, distinctive role of the virus itself or antiviral treatment remains to be clarified.

Hashimoto's thyroiditis patients need no treatment, because the disease is mostly asymptomatic, and often have small goitre [19]. Surgery is reserved for the patients who have significant pain, cosmetic, or pressure effects, after a trial of steroid therapy. Steroid therapy may result in resolution in some cases. If the patients have hypothyroidism, it needs thyroxine treatment [20].

## 4. Conclusion

Though a rare entity, thyroid tuberculosis should be considered in differential diagnosis of thyroid swelling presenting as abscess. FNAC and culture are the main diagnostic investigation. Sometimes, it may be associated with autoimmune thyroiditis. Treatment is mainly based on antituberculous drugs, but surgery is rarely required especially in cases presenting with large thyroid abscess.
